# Hepatoprotective effect of *Coreopsis tinctoria* flowers against carbon tetrachloride-induced liver damage in mice

**DOI:** 10.1186/s12906-017-1604-8

**Published:** 2017-03-04

**Authors:** Jen-Chieh Tsai, Chuan-Sung Chiu, Yun-Chieh Chen, Meng-shiou Lee, Xiu-Ying Hao, Ming-Tsuen Hsieh, Chun-Pin Kao, Wen-Huang Peng

**Affiliations:** 1 Department of Medicinal Botanicals and Health Applications, College of Biotechnology and Bio-resources, Da-Yeh University, 168 University Rd, Dacun, Chang-Hua, Taiwan, Republic of China; 2 Hsin Sheng College of Medical Care and Management, Taoyuan, Taiwan, Republic of China; 3 Department of Chinese Pharmaceutical Sciences and Chinese Medicine Resources, College of Pharmacy, China Medical University, Taichung, 404 Taiwan, Republic of China; 4 Institute of Microbiology, Xinjiang academy of Agricultural Sciences, Urumqi, Xinjiang China

**Keywords:** *Coreopsis tinctoria*, Hepatoprotective, Carbon tetrachloride

## Abstract

**Background:**

*Coreopsis tinctoria* is a traditional remedy for the management of various diseases including hepatitis. The hepatoprotective role of the plant is not scientifically explored till now. This study was designed to investigate the hepatoprotective potentials of the ethanol extract from *C. tinctoria* (CTEtOH) using an animal model of carbon tetrachloride (CCl_4_)-induced acute liver injury.

**Methods:**

CTEtOH (0.5 and 1.0 g/kg) and silymarin (200 mg/kg) were administered to the experimental mice for 7 days followed by 0.2% CCl_4_ (10 mL/kg of body weight (bw), ip), then all mice were sacrificed after 24 h. The serum alanine aminotransferase (ALT) and aspartate aminotransferase (AST) levels were measured. Histological analysis of liver was performed. The tumor necrosis factor-α (TNF-α), interleukin-1β (IL-1β), interleukin-6 (IL-6), nitric oxide (NO), malondialdehyde (MDA), and antioxidant enzymatic activities were also measured..

**Results:**

The results revealed that the serum ALT and AST levels significantly decreased after treatment with CTEtOH. Moreover, histological analyses indicated that CTEtOH (0.5 and 1.0 g/kg) and silymarin reduced the extent of CCl_4_-induced liver lesions. CTEtOH (0.5 and 1.0 g/kg) reduced the levels of malondialdehyde, nitric oxide, and proinflammatory cytokines (TNF-α and IL-1β). Furthermore, CTEtOH (1.0 g/kg) reduced the level of IL-6. The activities of antioxidant enzymes, namely superoxide dismutase and glutathione reductase, significantly increased after treatment with CTEtOH (0.5 and 1.0 g/kg) and that of glutathione peroxidase increased after treatment with 1.0 g/kg of CTEtOH.

**Conclusions:**

These results demonstrate the hepatoprotective effect of CTEtOH against CCl_4_-induced acute liver injury in mice, and the underlying hepatoprotective mechanisms are associated with antioxidant and antiproinflammatory activities.

## Background

*Coreopsis tinctoria* Nutt. (Asteraceae) is a small, glabrous, aromatic annual plant commonly used for folk medicinal purposes worldwide. In North American Indians, *C. tinctoria* is used to treat several disorders such as diarrhea, internal pain, and bleeding [1]. In addition, it has been used as a traditional remedy for diarrhea, liver diseases, and diabetes for a long period. Flower infusion tea is used as a beverage to treat hypertension and hyperlipidemia [2]. Pharmacological studies have indicated that *C. tinctoria* exhibits some biological effects including antioxidant [3], antidiabetic [4], antihypertensive [5], and cytoprotective [1] activities. Although *C. tinctoria* is used for liver diseases, studies have yet to report on its hepatoprotective effect. In addition, studies have identified rich flavonoids, such as chalcones, flavanones, and flavonols. Among these compounds, marein (okanin-4′-*O*-d-glucopyranoside) has been identified as a major flavonoid [2, 5].

The liver plays a vital role in metabolism and has some physiological functions [6]. Liver detoxification occurs in two phases, namely phase I (involving oxidation, reduction, and hydrolysis) and phase II (involving synthetic conjugations with sulfates, glucuronic acid, glutathione, acetate, and glycine), and these phases convert toxic materials into harmless metabolites and then excrete them from the body [7]. Liver diseases are considered serious problems which can be caused by toxic chemicals, drugs, and virus infiltration through ingestion or infection [8]. These toxins induce the production of reactive oxygen species (ROS), which can attack hepatic tissue and cause serious injury [9, 10].

Carbon tetrachloride (CCl_4_) is a potent toxin, and animal models involving CCl_4_-induced hepatic injury have been widely used to evaluate the potential of medicine or food to protect against hepatotoxicity [11]. The cytochrome P450 system metabolizes CCl_4_ to the highly reactive trichloromethyl radical CCl_3_‧, which can react with oxygen to form the trichloromethyl peroxyl radical CCl_3_OO‧ and then attack lipids or proteins. This reaction can initiate lipid peroxidation and cause damage to liver tissue [12]. Antioxidants may protect against oxidative stress-related liver pathologies such as CCl_4_-induced liver lesions by blocking the chain of lipid peroxidation [13]. In recent years, antioxidant activities of herbs have been comprehensively investigated and some herbs or derived chemical components are good sources [14].

This study investigated the hepatoprotective effect of the ethanol extract of *C. tinctoria* flowers (CTEtOH) on CCl_4_-induced acute liver damage in mice. Serum alanine aminotransferase (ALT) and aspartate aminotransferase (AST) levels were estimated to evaluate liver function. In addition, pathological biopsies were examined. The levels of proinflammatory cytokines such as TNF-α, IL-1β, and IL-6; nitric oxide (NO); malondialdehyde (MDA); and antioxidant enzymes such as superoxide dismutase (SOD), glutathione peroxidase (GPx), and glutathione reductase (GRd) were measured to elucidate the underlying mechanism. Silymarin, a group of flavones extracted from *Silybum marianum* L., is a strong antioxidant and an effective protective agent against CCl_4_-induced liver injury and hepatic fibrosis. In this study, silymarin was used as a positive control. The total polyphenol and total flavonoid contents were also determined.

## Methods

### Preparation of plant extract

*C. tinctoria* flowers were obtained and identified by Dr. Xiu-Ying Hao, Institute of Microbiology, Xinjiang Academy of Agricultural Sciences (Urumqi, Xinjiang, China). The plant specimen (voucher specimen number: CPSCMR-103-012) was deposited in the Department of Chinese Pharmaceutical Sciences and Chinese Medicine Resources, College of Pharmacy, China Medical University (Taichung, Taiwan). Dried flowers (3.0 kg) were soaked in 1 L of 70% ethanol at 45 °C four times and then filtered. The filtrates were concentrated under reduced pressure using a vacuum rotary evaporator. Subsequently, the remaining solution was lyophilized to obtain the crude extract (CTEtOH, 694.3 g). The extract was stored at −20 °C until further use.

### Materials

CCl_4_ was purchased from Merck Co. (Germany) and dissolved in olive oil (0.2%, v/v). AST, ALT, SOD, GPx, and GRd assay kits were purchased from Randox Laboratory Ltd (UK). TNF-α, IL-1β, and IL-6 were purchased from eBioscience Inc. (USA). Folin-Ciocalteu’s phenol reagent, sodium carbonate (Na_2_CO_3_), aluminum chloride (AlCl_3_), thiobarbituric acid, Griess reagent, catechin, rutin, and silymarin (≥90%) were purchased from Sigma-Aldrich Chemical Co. (USA). All other reagents used were of analytical grades.

### Animals

ICR mice (20–25 g) were obtained from BioLasco Taiwan Co., Ltd (Taipei, Taiwan). The mice were housed under normal laboratory conditions (21 ± 2 °C and 12/12-h light-dark cycle) with free access to standard pellet diet and water. All animal procedures were conducted in accordance with the standards set forth in the guidelines for the Care and Use of Experimental Animals by the Committee for the Purpose of Control and Supervision of Experiments on Animals and the National Institutes of Health. The study protocol was approved by the Animal Ethics Committee of China Medical University (Number: 103–314).

### CCl_4_-induced hepatotoxicity

The animals were randomly divided into six groups, with 10 mice in each group. Distilled water was administered to Group 1 (control) and Group 2 (CCl_4_) and silymarin (200 mg/kg) was administered to Group 3 orally. CTEtOH was administered to Groups 4, 5, and 6 at doses of 0.1, 0.5, and 1.0 g/kg (in 0.5% carboxymethylcellulose), respectively. After administration for 7 consecutive days, CCl_4_ (10 mL/kg BW, 0.2% in olive oil) was injected intraperitoneally 1 h after the last administration except for the control group. The mice in the control group were injected an equivalent volume of olive oil intraperitoneally. At 24 h after CCl_4_ injection, all the mice were sacrificed under anesthesia. Their livers were rinsed in four times volume of Tris-HCl buffer, then homogenized and centrifuged at 12,000 rpm for 10 min at 4 °C to separate the supernatant. The supernatant was used for histological analyses and TNF-α, IL-1β, IL-6, NO, MDA, and antioxidant enzymatic activity measurements.

### Serum biochemical analysis

Blood was collected and centrifuged at 3000 rpm (Beckman GS-6R, Germany) at 4 °C for 30 min to separate the serum. Serum ALT and AST levels were measured using spectrophotometric diagnostic kits (Roche, Germany).

### Histological analysis

Liver samples were fixed in a 10% buffered formaldehyde solution and processed using the paraffin slice technique. For hematoxylin and eosin staining, sections were stained with hematoxylin for 4 min, washed, and then stained with 0.5% eosin for an additional 4 min. The degree of liver damage was examined under a light microscope by a pathologist (Dr. Liao; Graduate Institute of Veterinary Pathobiology, Taichung, Taiwan, R.O.C.) who was blinded to the study purpose.

### MDA assay

MDA is the end product of lipid peroxidation. In this study, MDA was measured using the thiobarbituric acid-reactive substances (TBARS) assay [15]. One milliliter of the supernatant was mixed with 1 mL of TBA solution and boiled for 45 min to form red TBARS (under the acidic condition). The absorbance was measured at 532 nm. MDA levels are expressed in nanomoles per milligram of protein (nmole/mg protein).

### NO assay

NO levels were measured using the NO assay kit (ab65327) [16]. This kit provides an accurate and convenient measure of the total nitrate or nitrite concentration in a simple two-step process. In the first step, nitrate is converted to nitrite by nitrate reductase. In the second step, nitrite reacts with the fluorescent probe 2, 3-diaminonaphthalene, and the corresponding fluorescence is measured at Ex/Em = 360/450 nm. NaOH enhances the fluorescence yield. The fluorescence intensity is proportional to the total NO production. The kit has been tested with culture media, plasma, and tissue homogenates. The results are expressed as the sum of nitrite and nitrate concentrations.

### TNF-α, IL-1ß, and IL-6 assays

TNF-α, IL-1β, and IL-6 were measured using enzyme-linked immunosorbent assays. A capture antibody was added to each well and incubated overnight. On the following day, a biotinylated antibody was added and incubated with sample tissues or standard antigens. Finally, streptavidin was added to end the reaction, and the absorbance was recorded at 450 nm. The results are expressed in picograms per milligram of protein (pg/mg) for cytokine concentrations.

### Antioxidant enzymatic activity measurements

Liver homogenates were prepared in cold Tris-HCl buffer by using a homogenizer, to evaluate SOD, GPx, and GRd activities. These antioxidant enzymatic activities were determined using the kits purchased by Randox Laboratories Ltd. by following the manufacturer’s instructions and detected using the Chem Well®-T Automated Chemistry Analyzer. The results are expressed in units per milligram of protein (U/mg protein).

### Determination of total polyphenol and total flavonoid content

The total polyphenol content was evaluated using the Folin-Ciocalteu method [17]. The sample (20 μL, 250 μg/mL) was added to 40 μL of Folin-Ciocalteu reagent and 200 μL of distilled water in each well. The mixture was shaken and maintained at room temperature for 5 min before the addition of 40 μL of 20% Na_2_CO_3_. The solution was mixed, and the absorbance was measured at 680 nm. The assay was conducted in triplicates. (+)-Catechin was used for the calibration curve, and the results are expressed in micrograms of (+)-catechin equivalents per milligram of the dry weight extract.

The total flavonoid content was determined as follows. First, 100 μL of the sample extract was mixed with an equal volume of AlCl_3_ · 6H_2_O solution (2%). The mixture was shaken and maintained at room temperature for 10 min. Subsequently, the absorbance was measured at 430 nm [18]. The assay was conducted in triplicates. Rutin was used for the calibration curve, and the results are expressed in micrograms of rutin equivalents per milligram of the dry weight extract.

### HPLC analysis

The HPLC fringerprint was established according to previous study [2] and by a Shimazu HPLC system comprising of a LC-10 AD VP Pumps, a SPD-10AV VP Detector, aSIL-10 AD VP AutoSampler and a DGU - 14A Degasser. Separations were carried out with a Thermo Scientific Hypersil ODS C18 column (4.6 mm × 250 mm, 5 μm, USA). The injection volume was 10 μl and each sample was filtered through a 0.45 μm Minipore filter. The mobile phase consisted of acetonitrile (A) and 0.05% formic acid (B) using a gradient elution of 5–20% A at 0–60 min and 20–40% A at 60–110 min. The flow rate was 1.0 ml/min and the detection wavelength was 254 nm.

### Statistical analysis

All data are presented as the mean ± SEM. Statistical analyses were performed using SPSS software and one-way ANOVA followed by Scheffe’s multiple range tests. Histological analyses were conducted using the nonparametric Kruskal-Wallis test followed by the Mann-Whitney *U* test. The criterion for statistical significance was *p* < 0.05.

## Results

### Effect of CTEtOH on CCl_4_-induced hepatotoxicity

**Fig. 1:**
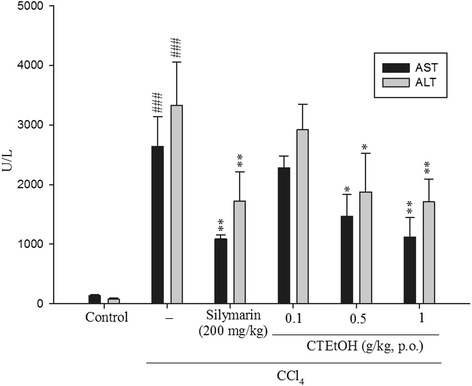
Effects of CTEtOH and silymarin on serum AST and ALT levels in mice treated with CCl_4_. Each value is represented as the mean ± SEM (*n* = 10). ^###^ *p* < 0.001, compared with the control group. *p < 0.05 and **p < 0.01, compared with the CCl_4_ group (one-way ANOVA followed by Scheffe’s multiple range test)

Figure 1 presents the hepatoprotective effect of CTEtOH on CCl_4_-induced liver injury. Serum ALT and AST levels increased in the CCl_4_ group significantly. However, these increased serum ALT and AST levels decreased after treatment with CTEtOH (0.5 and 1.0 g/kg) and silymarin (200 mg/kg). These results indicate that CTEtOH has protective functions against CCl_4_-induced liver damage.

### Histological analyses

**Fig. 2:**
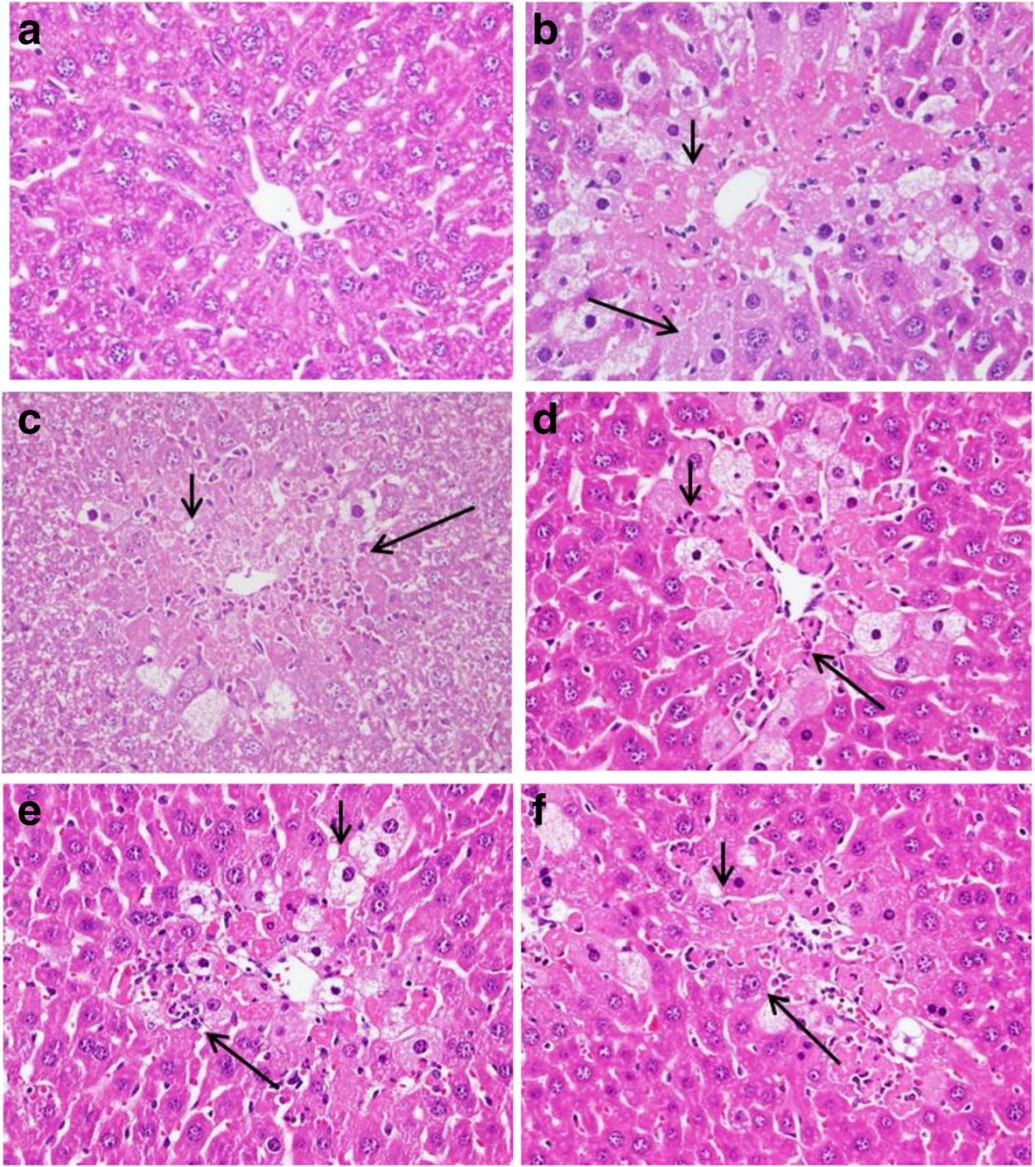
Hepatic histological analyses of CTEtOH and silymarin on CCl_4_-induced acute liver damage in mice. Liver tissues were subjected to hematoxylin and eosin staining (400×). **a** Control group; (**b**) animals treated with 0.2% CCl_4_; displayed cell necrosis (long arrow) and vacuole formation (short arrow) (**c**) animals pretreated with silymarin (200 mg/kg) and then treated with CCl_4_; (**d**–**f**) animals pretreated with CTEtOH (0.1, 0.5, and 1.0 g/kg) and then treated with CCl_4_

As illustrated in Fig. 2, CCl_4_-induced injury included increased vacuole formation, neutrophil infiltration, and necrosis. However, the damage decreased after pretreatment with CTEtOH (0.5 and 1.0 g/kg) and silymarin (200 mg/kg).

### Effect of CTEtOH on the MDA and NO levels

**Fig. 3:**
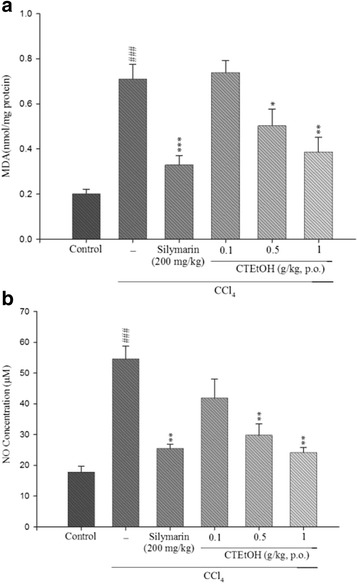
Effects of CTEtOH and silymarin on the liver MDA (**a**) and NO (**b**) levels in mice treated with CCl_4_. Each value is represented as the mean ± SEM (*n* = 10). ^###^ *p* < 0.001, compared with the control group. *p < 0.05, **p < 0.01, and ***p < 0.001, compared with the CCl_4_ group (one-way ANOVA followed by Scheffe’s multiple range test)

The MDA level was determined to evaluate the degree of hepatic lipid peroxidation. The results revealed that the MDA level significantly increased in the CCl_4_ group (Fig. 3a). However, the MDA level decreased in the CTEtOH (0.5 and 1.0 g/kg) and silymarin (200 mg/kg) groups. As presented in Fig. 3b, the NO level considerably increased in the CCl_4_ group. However, compared with the CCl_4_ group, the NO level significantly decreased in the CTEtOH (0.5 and 1.0 g/kg) and silymarin groups.

### Effect of CTEtOH on TNF-α, IL-1β, and IL-6 levels in the liver

Table 1 presents the levels of proinflammatory cytokines, namely TNF-α, IL-1β, and IL-6, in CCl_4_-induced acute liver injury. The levels of these cytokines in the CCl_4_ group were remarkably higher than those in the control group. Treatment with silymarin (200 mg/kg) or CTEtOH (0.5 and 1.0 g/kg) significantly reduced the levels of TNF-α and IL-1β. Similarly, treatment with silymarin (200 mg/kg) or CTEtOH (1.0 g/kg) significantly reduced the levels of IL-6.

**Table 1:** Effect of CTEtOH on the levels of different proinflammatory cytokines in CCl_4_-treated mice

Groups	Pro-inflammatory cytokines (pg/mg protein)		
	TNF-α	IL-1β	IL-6
Control	31.88 ± 2.17	161.86 ± 13.66	8.70 ± 0.51
CCl_4_	82.74 ± 5.02^###^	1608.68 ± 33.86^###^	33.89 ± 1.76^###^
CCl_4_ + Silymarin (200 mg/kg)	51.24 ± 2.04^***^	994.86 ± 35.66^***^	15.97 ± 1.60^***^
CCl_4_ + CTEtOH (0.1 g/kg)	87.54 ± 4.53	1445.95 ± 88.75	36.05 ± 5.77
CCl_4_ + CTEtOH (0.5 g/kg)	66.02 ± 6.78^*^	1275.45 ± 151.73^*^	28.28 ± 2.28
CCl_4_ + CTEtOH (1.0 g/kg)	60.82 ± 4.76^*^	1168.69 ± 97.21^**^	23.23 ± 1.30^*^

Each value is represented as the mean ± S.E.M. (*n* = 10). ^###^ *p* < 0.001, compared with the control group. *p < 0.05, **p < 0.01, and ***p < 0.001, compared with the CCl_4_ group (one-way ANOVA followed by Scheffe’s multiple range test)

### Effects of CTEtOH on antioxidant enzymatic activities

The activities of SOD, GPx, and GRd were measured to evaluate the antioxidant effects of CTEtOH. Compared with the control group, the activities of these antioxidant enzymes significantly decreased in the CCl_4_ group (Table 2). Furthermore, the activities of SOD and GRd significantly increased after treatment with CTEtOH at doses of 0.5 and 1.0 g/kg. The activity of GPx significantly increased after treatment with 1.0 g/kg of CTEtOH.

**Table 2:** Effect of CTEtOH on the activities of different antioxidant enzymes in CCl_4_-treated mice

Groups	SOD (U/mg protein)	GPx (U/mg protein)	GRd (U/mg protein)
Control	5.33 ± 0.12	0.856 ± 0.088	0.104 ± 0.012
CCl_4_	4.68 ± 0.11^##^	0.391 ± 0.061^###^	0.051 ± 0.007^##^
CCl_4_ + Silymarin (200 mg/kg)	6.06 ± 0.14^***^	0.812 ± 0.120^**^	0.092 ± 0.008^**^
CCl_4_ + CTEtOH (0.1 g/kg)	4.74 ± 0.18	0.436 ± 0.068	0.056 ± 0.040
CCl_4_ + CTEtOH (0.5 g/kg)	5.93 ± 0.08^***^	0.567 ± 0.052	0.801 ± 0.006^*^
CCl_4_ + CTEtOH (1.0 g/kg)	5.92 ± 0.07^***^	0.771 ± 0.091^**^	0.891 ± 0.011^**^

Each value is represented as the mean ± S.E.M. (*n* = 10). ^##^ *p* < 0.01 and ^###^ *p* < 0.001, compared with the control group. *p < 0.05, **p < 0.01, and ***p < 0.001, compared with the CCl_4_ group (one-way ANOVA followed by Scheffe’s multiple range test)

### Total polyphenol and flavonoid contents

The total polyphenol content was measured using the Folin-Ciocalteu method and is expressed in milligram gallic acid equivalents per gram of the dry weight extract (mg GAE/g). The results revealed that the total polyphenol content of CTEtOH was 248.14 ± 6.51 mg GAE/g. The total flavonoid content is expressed in milligram rutin equivalents per gram of the dry weight extract (mg RE/g) and the content of CTEtOH was 123.41 ± 4.53 mg QE/g.

### HPLC analysis

**Fig. 4:**
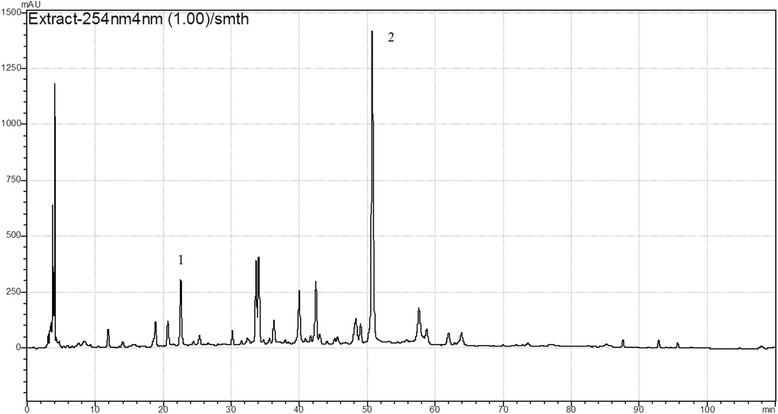
HPLC chromatogram at 254 nm. Key to the peaks: 1-chlorogenic acid, 2-marein

The HPLC fingerprint of CTEtOH is shown in Fig. 4. In the chromatogram, peaks at the retention time of 22.55 min and 50.71 min were detected, representing chlorogenic acid and marein respectively.

## Discussion

Animal models of CCl_4_-induced liver injury are commonly used to investigate the hepatoprotective effects of natural medicine. An increase in serum AST and ALT levels has been attributed to the damaged hepatic structure. Liver injury can be evaluated by determining serum AST and ALT levels [19]. The results of the present study reveal that treatment with CTEtOH significantly reduced AST and ALT levels. In addition, histological analyses indicate that hepatic cell injury was accompanied by vacuole formation, neutrophil infiltration, and necrosis around the central vein after CCl_4_ administration. The observed liver damage was significantly improved after treatment with CTEtOH. These results indicate the hepatoprotective effect of CTEtOH on CCl_4_-induced hepatotoxicity.

Inflammation is initiated by CCl_4_-induced hepatotoxicity, followed by the release of proinflammatory mediators including inducible NO synthase (iNOS), TNF-α, IL-1β, and IL-6. NO is a crucial proinflammatory mediator and a highly active nitrogen species produced by iNOS during the conversion of l-arginine to l-citrulline [20]. Moreover, NO reacts with superoxide anions to form a strong cytotoxic oxidant, namely peroxynitrite, which causes lipid peroxidation and cellular damage [21]. The overproduction of NO can cause hepatic injury, and the inhibition of NO can reduce inflammatory damage. TNF-α, IL-1β, and IL-6 are critical cytokines in inflammatory responses, and their levels are increased during the development of liver damage [13]. TNF-α, which is produced by Kupffer cells, can induce immune responses by activating T cells and macrophages and further stimulate the secretion of other inflammatory cytokines and the production of NO [22]. IL-1β is another proinflammatory cytokine playing a vital role in the regulation of hepatic NO synthesis [23]. IL-6 stimulates hepatocytes to produce various acute-phase proteins and protects against liver injury [24]. Our results reveal that the production of NO, TNF-α, IL-1β, and IL-6 significantly decreased after treatment with CTEtOH, thus demonstrating that CTEtOH exerts a hepatoprotective effect on CCl_4_-induced injury.

Lipid peroxidation is one of the major characteristics of CCl_4_-induced hepatotoxicity [25]. MDA, the end product of lipid peroxidation, is formed by free radicals attacking the plasma membrane and is widely used as a marker of lipid peroxidation injury [26]. In the current study, an increase in the hepatic MDA level suggested the enhancement of lipid peroxidation, consequently leading to hepatic damage as well as the inactivation of the antioxidant defense system. However, the increased hepatic MDA level decreased after treatment with CTEtOH. ROS formed in the CCl_4_-induced liver damage model could significantly reduce the expressions of antioxidant enzymes such as SOD, GPx, and GRd [19]. Therefore, an increase in antioxidant activity and the inhibition of free radical generation are positively correlated with hepatic protection. SOD dissimulates superoxide to H_2_O_2_ in the enzymatic defense against oxygen toxicity. Both GPx and GRd are GSH-related enzymes and play detoxifying and antioxidant roles in cellular defense through conjugation with glutathione or the reduction of free radicals. GPx works with GSH to metabolize H_2_O_2_, a harmful toxin, to nontoxic products, and GRd catalyzes the reduction of GSSG to GSH [27]. Our results reveal that the activities of SOD, GPx, and GRd significantly decreased during the development of CCl_4_-induced acute liver injury; this finding is in agreement with those of previous studies [11, 28]. In this study, the activities of SOD, GPx, and GRd improved after treatment with CTEtOH. These findings suggest that CTEtOH reduces ROS production by increasing hepatic antioxidant activities and protecting against hepatotoxicity. The suppression of MDA production is likely to promote the activities of SOD, GPx, and GRd. Furthermore, an increase in SOD activity not only increases the superoxide anion scavenging capacity but also prevents the peroxynitrite production.

Phenolic compounds, such as flavonoids and phenolic acids, that are present in many plant species were reported to express high antioxidant activity in stabilizing lipid oxidation [29]. These phenolic compounds possess antioxidant properties and protect hepatocytes against chemically induced liver carcinogenesis. The mechanisms underlying this chemoprevention include stimulation of the induction of apoptosis in transformed hepatocytes and inhibition or scavenging of ROS, which eliminates or lowers the extent of lipid peroxidation or inflammation in liver cells, ultimately preventing the incidence of hepatic necrosis [30]. In addition, the antioxidant properties of some of these plants, especially of their isolated compounds, have therapeutic benefits in pathological conditions such as liver diseases, cancer, diabetes, and heart diseases. However, a considerable percentage of these potential antioxidant plants, particularly those with promising hepatoprotective properties, is yet to be explored. *C. tinctoria* contains various types of bioactive chemical compounds, such as flavonoids, phenols, phenylpropanoids, polyacetylene glycosides, and sterols, and flavonoids are major phytochemical compounds [5]. Some chemical compositions of *C. tinctoria* have been separated and identified, including marein, okanin, coreopsin, taxifolin, and isookanin [2, 5]. In the present study, the results indicated that *C. tinctoria* contains high contents of total polyphenols and total flavonoids. The HPLC fingerprint also indicated that *C. tinctoria* have chlorogenic acid, which displays good hepatoprotective effect [31]. However, additional studies should be conducted to determine the principal active compounds and their mechanisms underlying the hepatoprotective effect.

## Conclusions

In conclusion, this is the first study to demonstrate that CTEtOH expressed hepatoprotective activity against CCl_4_-induced acute hepatotoxicity in mice. The results indicate that CTEtOH not only enhances hepatic antioxidant enzyme activities and inhibits lipid peroxidation but also suppresses inflammatory responses in CCl_4_-induced liver damage. The possible hepatoprotective mechanism correlates with the inhibition of lipid peroxidation through increasing the activities of antioxidant enzymes and the regulation of proinflammatory mediators to maintain the integrity of hepatic cells. Because *C. tinctoria* is demonstrated in this study to exhibit a hepatoprotective effect against chemically induced liver injury, it can be potentially developed into a functional food or even a pharmacological agent for the prevention of liver diseases.

## Abbreviations

CTEtOH: The 70% ethanol extract of *Coreopsis tinctoria*
CCl_4_: Carbon tetrachloride
ALT: Alanine aminotransferase
AST: Aspartate aminotransferase
TNF-α: Tumor necrosis factor-α
IL-1β: Interleukin-1β
IL-6: Interleukin-6
NO: Nitric oxide
MDA: Malondialdehyde
SOD: Superoxide dismutase
GPx: Glutathione peroxidase
GRd: Glutathione reductase
iNOS: Inducible nitric oxide synthase
